# Pharmacokinetics of piperacillin/tazobactam in cancer patients with hematological malignancies and febrile neutropenia after chemotherapy

**DOI:** 10.1186/2050-6511-14-59

**Published:** 2013-11-28

**Authors:** José C Álvarez, Sonia I Cuervo, Javier R Garzón, Julio C Gómez, Jorge Augusto Díaz, Edelberto Silva, Ricardo Sánchez, Jorge A Cortés

**Affiliations:** 1 Universidad Nacional de Colombia, Facultad de Medicina, Bogotá, Colombia; 2 (GREICAH): Grupo de Investigación en Enfermedades Infecciosas en Cáncer y alteraciones hematológicas, Bogotá, Colombia; 3 Instituto Nacional de Cancerología, Bogotá, Colombia; 4 Departamento de Farmacia, Facultad de Ciencias, Universidad Nacional de Colombia, Bogotá, Colombia; 5 Departamento de Medicina, Facultad de Medicina, Ciudad Universitaria, Carrera 30 No. 45-03, edificio 471, oficina 510, Bogotá, A. A. 14490, Colombia

**Keywords:** Pharmacokinetics, Piperacillin/tazobactam, Beta-lactam antibiotics, Neutropenia, Fever, Chemotherapy, Hematological malignancies

## Abstract

**Introduction:**

Patients with febrile neutropenia (FN) exhibit changes in extracellular fluid that may alter the plasma concentrations of beta-lactams and result in therapeutic failure or toxicity. We evaluated the pharmacokinetics of piperacillin/tazobactam in patients with hematological malignancies and FN after receiving chemotherapy at a primary public cancer center.

**Methods:**

This was an open, nonrandomized, observational, descriptive, and prospective study. Samples from 15 patients with hematological malignancies and FN were evaluated after the administration of chemotherapy. Five blood samples were taken from each patient when the antibiotic level was at steady-state 10, 60, 120, 180, and 350 min after each dose. Antibiotic concentrations were measured using gel diffusion with *Bacillus subtilis*. All study participants provided written informed consent.

**Results:**

We investigated the pharmacokinetics of piperacillin in 14 patients between the ages of 18 years and 59 years and with a mean absolute neutrophil count of 208 cells per mm^3^ (standard deviation (SD) ± 603.2). The following pharmacokinetic measurements were obtained: maximum concentration, 94.1–1133 mg/L; minimum concentration, 0.47–37.65 mg/L; volume of distribution, 0.08–0.65 L/kg (mean, 0.34 L/kg); drug clearance (CL), 4.42–27.25 L/h (mean, 9.93 L/h); half-life (t_1/2_), 0.55–2.65 h (mean, 1.38 h); and area under the curve, 115.12–827.16 mg · h/L.

**Conclusion:**

Patients with FN after receiving chemotherapy exhibited significant variations in the pharmacokinetic parameters of piperacillin compared with healthy individuals; specifically, FN patients demonstrated an increase in t_1/2_ and decreased CL.

## Background

Variations in the pharmacokinetic (PK) and pharmacodynamic (PD) parameters of hydrophilic antimicrobial agents have been described previously in different pathological conditions (e.g., sepsis, trauma, burns, hypoalbuminemia), in animal models and in clinical trials evaluating febrile neutropenia (FN). These variations were identified because the extracellular fluid (ECF) levels in these patients were different than in healthy patients. Changes in ECF levels affect the plasma concentrations of antibiotics [1,2].

Beta-lactams are the first-choice antibiotics for the treatment of FN after chemotherapy, and inadequate serum levels of these antibiotics may lead to therapeutic failure. Cefepime, piperacillin/tazobactam, imipenem/cilastatin, and meropenem are beta-lactam antibiotics that exhibit activity against *Pseudomonas aeruginosa*, and these antibiotics are recommended as monotherapy for the empirical treatment of FN after chemotherapy [3].

Little information is available on the kinetic behavior of piperacillin or the piperacillin/tazobactam combination in cancer patients. However, addition of piperacillin or amikacin to moxalactam administration in FN patients has been shown to alter PK parameters compared with healthy individuals [4], and a randomized clinical trial evaluating PK parameters demonstrated a decrease in the volume of distribution (Vd) and drug clearance (CL) and an increase in half-life (t½) [5].

We investigated the PK parameters of piperacillin in Latin American patients with hematological malignancies and FN after receiving chemotherapy.

## Methods

We conducted an open, nonrandomized, observational, descriptive, and prospective study in the Hematology Department of the National Cancer Institute (NCI) in Bogota, Colombia. The Ethics Committees of the National University of Colombia and NCI approved the protocol. All study participants provided written informed consent.

### Patients

The inclusion criteria were patients: aged >18 years with a *de novo* diagnosis or recent diagnosis of hematological malignancy; currently undergoing chemotherapy; who had FN after receiving chemotherapy; did not have renal or liver failure; being treated with piperacillin/tazobactam (4.5 g, i.v.) every 6 h in the event of FN. Pregnancy tests were negative for women of reproductive age.

The exclusion criteria were patients: with chronic kidney disease defined as a estimated glomerular filtration rate (eGFR) <60 mL/min/1.73 m^2^[6]; with liver failure defined as Child–Turcotte–Pugh classes B or C [7]; who received combined antimicrobial therapy due to a polymicrobial bloodstream infection.

### Definitions

Neutropenia was defined as an absolute neutrophil count <1,000 cells per mm^3^ or in cases where a count <1,000 cells per mm^3^ was anticipated 3–5 days after chemotherapy. Neutropenia was also declared in cases of acute leukemia with a neutrophil count with a blast percentage >90%.

Fever was defined as a temperature of 38°C for 1 h or one temperature measurement >38.3°C. FN was secondary to intensive antineoplastic induction, re-induction, or maintenance chemotherapy.

### Laboratory tests

Five samples were taken from each of the 15 patients 10, 60, 120, 180, and 350 min after the dose of piperacillin/tazobactam attained a steady state. Piperacillin/tazobactam was administered as a generic product (Vitalis^®^) purchased by the institution for use in all patients. The piperacillin content in samples from different batches and patient sera was measured using a biological gel diffusion assay with *Bacillus subtilis* ATCC 6633. The following PK parameters were calculated from the concentration–time plots: maximum concentration (C_max_), minimum concentration (C_min_), Vd, CL, t½, and the area under the curve (AUC_0–∞_).

### Statistical analyses

STATA IC^®^ ver11.1 software was used for analyses. Two-tailed Student’s *t*-tests were used to compare independent samples at a significance defined as p < 0.05. PK parameters were compared between group of patients with albumin levels >3 g/dL*vs*. those with <3 g/dL, those who received a piperacillin/tazobactam in bolus dose, and those who received chemotherapy with Hyper-CVAD *vs*. another chemotherapy regimen (for Hyper-CVAD chemotherapy, course A comprised cyclophosphamide, vincristine, doxorubicin and dexamethasone, and course B consisted of methotrexate and cytarabine).

## Results

Originally, the study cohort was 15 patients. However, 1 patient (patient 003) was excluded because the inhibition zone was not clearly visible due to serum hyperviscosity and the white blood cell (WBC) count was 262,400 cells per mm^3^. The ages of the 14 studied patients ranged from 18 years to 59 years (mean, 31.9 years; standard deviation (SD), 15.4), and 8 of the patients were women. The albumin values recorded were between 2.1 g/dL and 4 g/dL (mean, 3.04 g/dL; SD, 0.56), and the neutrophil count was 0–2,380 cells per mm^3^ (mean, 208 cells/mm^3^; SD, 603.2). In 71.4% of patients, the Hyper-CVAD chemotherapy regimen was used alone or in combination with rituximab or imatinib (Table 1).

**Table 1:** General characteristics of patients

**Parameter**	**Number (%)**
**Age (years)**	
18– 24	7 (50.0)
25 - 31	3 (21,4)
40 - 60	4 (28.6)
**Sex**	
Female	8 (57.1)
Male	6 (42.9)
**Weight (kg)**	
40 - 50	5 (35.7)
51 – 60	6 (42.9)
61 - 80	3 (21.4)
Mean ± SD = 56.4 ± 11.2	
**Height (cm)**	
150 – 160	9 (64.3)
161 – 170	4 (28.6)
171 – 180	1 ( 7.1)
**BMI kg/m**^**2**^	
17.0 – 22.0	8 (57.2)
22.1 – 27.0	5 (35.7)
27.1 – 32.0	1 (7.1)
Mean ± SD = 21.9 ± 4.0	
**Creatinine clearance (mg/mL)**	
80 - 110	3 (21.4)
111 - 140	6 (42.9)
141 - 170	5 (35.7)
**Albumin g/dL**	
2.0 – 2.6	3 (21.4)
2.7 – 3.3	7 (50.0)
3.4 – 4.0	4 (28.6)
Mean ± SD = 3.0 ± 0.6	
**Antibiotic given in the previous month?**	
Yes	5 (35.7)
No	9 (64.3)
**Malignancy**	
Lymphoma	2 (14.3)
Lymphoid leukemia	9 (64.3)
Myeloid leukemia	3 (21.4)
**Number of neutrophils/mm**^**3**^	
Range	0–2380
Mean ± SD	208(603.2)
**Therapy cycles**	
1 – 2	6 (42.9)
3 – 5	3(21.4)
6 - 8	5 (35.7)
**FN-associated chemotherapy**	
Hyper-CVAD	10 (71.4)
No Hyper-CVAD	4 (28.6)

BMI, body mass index; FN, febrile neutropenia; GFR, glomerular filtration rate.

For Hyper-CVAD chemotherapy, course A comprised cyclophosphamide, vincristine, doxorubicin and dexamethasone, and course B consisted of methotrexate and cytarabine.

Mentioned on page 6.

Concentrations (μg/mL) *versus* time were plotted for each patient to generate an exponential curve, which was used to calculate PK parameters. Visual inspection revealed one-compartment model behavior of piperacillin/tazobactam. Table 2 presents the PK parameters for each patient.

**Table 2:** PK parameters for each patient

**Patient**	**C**_**max **_**(μg/mL)**	**C**_**min **_**(μg/mL)**	**VD (L/kg)**	**Ke (h**^**–1**^**)**	**CL (L/h)**	**t**_**1/2 **_**(h)**	**AUC [0-inf]**	**Dose in mg/kg**
1	283.97	13.29	0.26	0.53	7.45	1.31	398.78	54.05
2	200.56	16.87	0.35	0.44	8.70	1.59	419.14	52.63
4	337.21	6.36	0.22	0.69	8.15	1.01	232.15	70.18
5	211.53	18.76	0.35	0.43	8.19	1.60	446.61	46.51
6	153.13	18.74	0.61	0.36	9.30	1.95	311.08	85.11
7	97.37	3.88	0.52	0.57	23.54	1.21	163.28	63.49
8	94.10	2.22	0.65	0.64	27.25	1.08	115.12	58.82
10	465.78	13.64	0.15	0.57	4.93	1.21	607.15	86.96
11	162.34	5.18	0.42	0.53	13.13	1.30	317.32	66.67
12	179.52	37.65	0.45	0.26	5.84	2.65	418.48	60.61
13	318.81	28.34	0.26	0.44	5.48	1.59	667.45	62.50
14	319.55	19.07	0.30	0.50	6.20	1.40	477.15	78.43
15	513.74	4.52	0.10	0.82	6.38	0.85	678.99	86.96
16	1133	0.47	0.08	1.25	4.42	0.55	827.16	66.67
**Mean**	319.33	13.50	0.34	0.57	9.93	1.38	434.28	67.11
**σ(n-1)**	266.08	10.71	0.18	0.24	6.95	0.51	205.17	13.01

Mentioned on page 6.

Statistically significant differences in PK parameters were evaluated by comparing the albumin values (Table 3) in patients with levels <3 g/dL and >3 g/dL and the use of Hyper-CVAD chemotherapy against other regimens.

**Table 3:** PK parameters in patients with albumin >3 g/dL and <3 g/dL with statistically significant differences

**PK parameter**	**Albumin <3 g/dL**	**Albumin >3 g/dL**	**Student’s *t*-test**	**P**
**C**_**min **_**(μg/mL)**	8.3	20.4	4.5135	0.0305
**Ke (h**^**–1**^**)**	0.7	0.4	-2.4515	0.0486
**t**_**1/2 **_**(h)**	1.1	1.7	-2.5709	0.0245

Mentioned on pages 6 and 8.

## Discussion

The present study confirmed previous observations of differences in the PK parameters of piperacillin/tazobactam in patients with FN and cancer. Patients with cancer and FN after chemotherapy are prone to cachexia, hypoalbuminemia, and the development of a third space [2]. Furthermore, the high protein content in exudates favors drug binding to proteins, which slows distribution of the drug in the systemic circulation, decreases C_max_ and increases t_1/2_.

The current study, in which piperacillin/tazobactam was administered as a bolus dose every 6 h, confirmed that the C_min_, elimination rate (Ke), and t_1/2_ parameters of piperacillin/tazobactam were modified in patients whose albumin levels were <3 g/dL. However, patients with an albumin level <3 g/dL would be expected to exhibit a higher free faction of antibiotic and a higher CL because these two parameters are directly related (renal CL = unbound drug × eGFR) [8]. Indeed, a difference in GFR was observed between subgroups because this rate increased in patients with albumin levels > 3 g/dL. However, other statistically significant differences between these groups were not observed. Organ function can be diminished in patients with lower albumin levels. For example, kidney function may increase tumor progression or decrease functional reserve, and non-renal CL or other non-quantified clinical variables may also be altered.

Table 4 summarizes the values of the means and SD for the calculated PK parameters and other patient characteristics in comparison with the work of Drusano et al. [4], Drusano et al. [5] and Mattoes et al. [9].

**Table 4:** Summary of PK parameters for piperacillin/tazobactam as compared with other studies

**Parameter**	**Present study**	**Strayer et al.**	**Shea et al.**	**Auclair et al.**	**Mattoes et al.**	**Langaartner et al.**	**Drusano et al. 1985**	**Drusano et al. 1989**
**C**_**max **_**(μg/mL)**	319.3 ± 266.08	366.7	108.2 ± 31.7	SD	282.2 ± 57.7	231 ± 66	152	SD
**C**_**min **_**(μg/mL)**	13.5 ± 10.71	SD	27.6 ± 26.3	SD	SD	11.5 ± 14.8	12	SD
**VD (L/kg)**	0.34 ± 0.18	SD	0.28 ± 0.07	0.12	0.2	34.6*	0.21 ± 0.14	0.13 ± 0.03
**Ke (h**^**–1**^**)**	0.57 ± 0.24	SD	SD	SD	0.86 ± 0.09	SD	SD	SD
**CL (L/h)**	9.93 ± 6.45	SD	8.6 ± 3.0	7.8	10.9 ± 2.5	10.23	8.31 ± 3.39	6.1 ± 2.54
**t**_**1/2 **_**(h)**	1.38 ± 0.51	1.3	2.1 ± 1.2	0.9	0.81 ± 0.08	2.4 ± 1.2	1.47 ± 0.95	1.54 ± 0.42
**AUC**_**[0–∞) **_**mg•h/L**	434.28 ± 205.17	488.9	527.5 ± 216.1	290	380.4 ± 72.6	391 ± 183	635.3 ± 253.2	SD
**Weight (kg)**	56.4 ± 26.78	*In vitro*	79.6 ± 13.8	SD	69.8 ± 15.7	60 - 86	69.5	70.2 ± 29.05

*Data shown in L.

Mentioned on pages 7, 8 and 9.

Analyses of the piperacillin/tazobactam concentration against time plots revealed a one-compartment model, as described previously in an *in vitro* study by Strayer et al. [10]. The PK parameters of piperacillin alone at 3 g every 6 h were compared with those of 3 g piperacillin every 6 h or 4 g every 8 h in combination with 0.375 g or 0.5 g tazobactam, respectively (Table 3). The PK parameters in our FN patients were related directly to higher Vd and CL values, which explains the similar t_1/2_ value as that observed by Strayer et al. (Table 4) [10].

A one-compartment model of behavior was observed previously when piperacillin/tazobactam was administered as a prolonged infusion to 13 patients who were hospitalized for the treatment of an infectious process [11]. Of these patients, 6 were noncritical and 7 were critical, and they received 4.5 g of piperacillin/tazobactam as a 4-h intravenous infusion every 8 h. These previous results by Shea et al. [11] suggested that the lower t_1/2_ value was due to an increase in CL, despite a similar Vd, for PK parameters (Table 4) [11].

Piperacillin doses between 8 g per day and 18 g per day were administered with tazobactam to four groups of healthy patients. Auclair et al. found that one-compartment model behavior was determined by visual inspection of the logarithmic relationship of plasma and urinary concentrations against time [12]. Piperacillin elimination is accomplished primarily *via* active tubular secretion by the kidney, but elimination is assisted by glomerular filtration and biliary excretion. Saturation of these mechanisms at plasma concentrations of piperacillin/tazobactam is unlikely. A nonlinear elimination, as explained by the Michaelis–Menten equation, would occur in the event of saturation, which would require a different PK analysis (Table 4) [12]. The filtration rate of a drug and the subsequent elimination rate are dependent upon the volume of the glomerular filtrate and free drug concentrations in the plasma (Table 4) [13].

One limitation of this study was the lack of a standardized method for administration of piperacillin/tazobactam by nurses with regard to the reconstitution of the powder forms of the drugs, solvent volume, or rate of infusion of the diluted antibiotic. This variability underlies representation of the concentration–time plots for antibiotic administration as an infusion in some patients and a bolus in others. Therefore, calculations of PK parameters were undertaken using interpretations of these two modes of drug administration. Mattoes et al. [10] investigated 12 healthy adults who received piperacillin/tazobactam (4.5 g, i.v.) every 6 h and demonstrated that patients with FN exhibited higher values of Vd and lower values of CL. These results may explain the lower values of Ke and higher values of t_1/2_ as compared with healthy individuals. PK parameters were investigated in patients in the intensive care unit (ICU) after administration of a 4-g bolus of piperacillin every 8 h or a 4-g bolus with subsequent continuous 8-g infusion for 24 h. PK parameters in patients who received a bolus administration compared with patients with FN in our study exhibited a similar value of CL and lower value of Vd, which would explain the lower t_1/2_ value compared with ICU patients. Langgartner et al. found that PK parameters often differed in healthy subjects because a previous study found that an increase in t_1/2_ and decrease in CL were due to decreased clearance of creatinine (Table 4) [14].

In another study, moxalactam was administered to FN patients and piperacillin or amikacin were included randomly. Drusano et al. measured the following PK parameters for piperacillin: Vd = 0.21 (SD = 0.14) L/kg; CL = 8.31 (SD 3.39) L/h/1.73 m^2^; and t_1/2_ = 1.47 (SD = 0.95) h (Table 4) [4]. Furthermore, Drusano et al. conducted a randomized, double-blind clinical trial in which one group received piperacillin and amikacin and the other group received imipenem/cilastatin. PK parameters were measured and decreases were found in Vd (0.13 (SD = 0.03) L/kg) and CL (6.1 (SD = 2.54) L/h/1.73 m^2^) and an increase in t_1/2_ (1.54 (SD = 0.42) h) noted [5].

## Conclusions

We found that piperacillin/tazobactam exhibited a one-compartment model of PK behavior in neutropenic patients. These data are in accordance with those of previous reports.

FN patients after chemotherapy exhibited important variations in PK parameters compared with healthy individuals. FN patients exhibited an increase in t_1/2_ and a decrease in CL.

FN patients should receive piperacillin/tazobactam doses based on weight (75 mg/kg) due to the low weight of these FN patients and the observed variations in plasma concentrations.

### Study limitations

One important limitation of this study was the inability to standardize the infusion rate or the amount of piperacillin/tazobactam diluent. This variation caused the values of plasma concentrations of antibiotics to differ from the expected values over time in some patients. This problem necessitated evaluation of the exponential curves from which the PK parameters were calculated with fewer samples than were taken. Serum sample measurements for piperacillin/tazobactam corresponded to those for different batches from the same drug company, but the piperacillin content in one sample from a different batch was not significantly different (results to be published).

## Abbreviations

ECF: Extracellular fluid; FN: Febrile neutropenia; ICU: Intensive care unit; NCI: National cancer institute; PD: Pharmacodynamic; PK: Pharmacokinetic.

## Competing interests

The authors declare that they have no competing interest.

## Authors’ contribution

JCA collaborated on data collection, PK calculations, and drafting of the manuscript. SIC participated in the design and preparation of the manuscript and coordinated the research at the research site. JRG presented the research idea and designed the research protocol. JCG collaborated on the selection and enrolment of patients, project management at the NCI, and data analyses. ES carried out the biological tests and laboratory work. JAD assisted in PK analyses. RS undertook the statistical analyses. JAC assisted in protocol development and data analyses. All of the authors approved the final manuscript and agreed upon its submission to *BMC Pharmacology and Toxicology*.

## Pre-publication history

The pre-publication history for this paper can be accessed here:

http://www.biomedcentral.com/2050-6511/14/59/prepub
